# Evaluation of the revised sense of coherence scale in a representative German sample

**DOI:** 10.1371/journal.pone.0209550

**Published:** 2018-12-31

**Authors:** Myriam V. Thoma, Shauna L. Mc Gee, Jörg M. Fegert, Heide Glaesmer, Elmar Brähler, Andreas Maercker

**Affiliations:** 1 Psychopathology and Clinical Intervention, Institute of Psychology, University of Zurich, Zurich, Switzerland; 2 University Research Priority Program “Dynamics of Healthy Aging”, University of Zurich, Zurich, Switzerland; 3 Department of Child and Adolescent Psychiatry and Psychotherapy, University of Ulm, Ulm, Germany; 4 Department of Medical Psychology and Medical Sociology, University of Leipzig, Leipzig, Germany; 5 Department of Psychosomatic Medicine and Psychotherapy, University Medical Center of the Johannes Gutenberg University Mainz, Mainz, Germany; University of Belgrade, SERBIA

## Abstract

**Background and objectives:**

To evaluate the Revised Sense of Coherence (SOC-R) scale in a large representative German sample.

**Design:**

A nationwide household survey involving a total of 2510 face-to-face interviews.

**Methods:**

In addition to the SOC-R, childhood trauma and maltreatment (CTM), lifetime traumatic events (Childhood Trauma Questionnaire, CTQ, and the Life Events Checklist for DSM-5, LEC-5), and mental health (Patient Health Questionnaire, PHQ-4) were assessed.

**Results:**

The final sample consisted of *N* = 2373 participants (52.3% females; *M* = 48.24 years). Confirmatory factor analyses confirmed a three-factor structure for the SOC-R (‘manageability’, ‘balance’, ‘reflection’) with acceptable indices (RMSEA .066; 90% CI [.062, .071]). Reliability analyses revealed good internal consistency (α = .87). Construct validity was supported by significant but low correlations with psychopathology. Gender marginally influenced SOC-R (*t* = 1.99, *p* = .05). Moderation analyses revealed that SOC-R exerted a protective impact on depression in the context of CTQ (*t* = 2.29, *p* < .05) and lifetime traumatic events (*t* = –2.37, *p* < .05).

**Conclusions:**

This study supports the psychometric properties of the SOC-R and emphasizes the importance of considering salutogenic effects to better understand interindividual differences in the effect of adversity.

## Introduction

Given the fact that the experience of adversities is an inevitable aspect of human life, it is crucial to understand why individuals differ in their responses to adversity. The Sense of Coherence (SOC) construct, originally developed by Antonovsky [1], explains interindividual differences in response to adversity (see also [2]). It is based upon the assumption that the experience and successful handling of adversity is an indispensable prerequisite for the development of psychological resistance, which assists in maintaining or promoting health [3]. The corresponding psychometric scale [4] has received much empirical attention and has become one of the most frequently applied salutogenic instruments across the globe [5]. While some studies have found the SOC instrument to be a reliable and valid measure for assessing sense of coherence [6], other studies have criticized its psychometric properties and measurement [7]. More specifically, this criticism has involved its factorial structure [8, 9], external validity [10, 11], stability [12, 13], as well as its utility [14].

Bachem and Maercker [14] proposed the revised SOC (SOC-R) scale that addressed the aforementioned shortcomings of the original scale. Conceptually, the definition of the SOC-R refers to “… the general ability to perceive life phenomena as connected to each other and to balance positive and negative appraisals of life experiences” [14]. It may thus be understood as a meta-heuristic, comparable, for instance, to the interactive concept of (stress-related) resilience. Resilience can be defined as an ability to adapt to (or reduced vulnerability to) stress or adversity [15]. Resilience is a common phenomenon [16], that can be described as “..an interactive concept that is concerned with the combination of serious risk experiences and a relatively positive psychological outcome despite those experiences” [17] as well as “..the ability to maintain a stable equilibrium” [18]. The SOC-R is particularly dedicated to the acknowledegement of the co-existence and integration of positive and negative life experiences by respecting and accepting those as equivalent facets of life.

The SOC-R has recently been validated in various samples differing in the degree of experienced adversities and age. For its initial validation, Bachem and Maercker [14] examined two middle-aged samples: one sample (from now on referred to as Sample 1) consisted of *N* = 334 bereaved participants (*M*_AGE_ = 44 years, 87.4% women) who had lost someone close to them in the recent past. The other sample (i.e. Sample 2) consisted of *N* = 157 control participants (*M*_AGE_ = 40 years, 63.1% women). With respect to the factorial structure, the conducted confirmatory factor analyses (CFA) proposed a three-factor solution for both samples, comprising the dimensions ‘manageability’, ‘reflection’, and ‘balance’. The model fit indices were better for Sample 1 in comparison to Sample 2 [14]. This was interpreted as an indication of a higher relevance of the SOC-R for individuals dealing with major (as opposed to minor) life events. Reliability analyses revealed that the SOC-R scale can be considered internally consistent and stable over the observed time intervals of one and 15 months. Convergent and discriminant validity was tested by correlating the SOC-R with commonly applied mental health measures (e.g. depression, anxiety, optimism, and posttraumatic growth) and were found to be good. On the basis of this initial validation of the revised SOC scale, it was cautiously concluded that the SOC-R scale had overcome the methodological shortcomings of the original scale [14].

In a next step, in order to evaluate the psychometric properties of the SOC-R in an older adult sample (i.e. Sample 3), we examined the SOC-R in *N* = 268 Swiss participants (Mage = 67 years, 71.3% women, [19]). The sample was divided into a younger (50–64 years) and an older (65 years and older) group to test whether the SOC-R may also be of relevance for individuals at differing older-age stages. The three-factor model was replicated with a good model fit in the complete sample as well as in both age-group samples [19]. Internal consistencies for two out of three scales were good (i.e. low internal consistency was shown for the ‘balance’ subscale). Convergent and discriminant validity were meaningful and within an acceptable range. Age was not shown to meaningfully impact SOC-R, implying stability of the SOC-R measure (however, keeping in mind that this was a cross-sectional study and therefore not able to address the question with regard to intraindividual stability over time). This study tentatively suggests that the SOC-R scale also seems to be a reliable and valid measure for assessing sense of coherence in adults of advanced age [19].

The construct of the SOC is closely linked to the experience of adversity. On one hand, it is theoretically thought to develop through the successful coping with adversity [4]. On the other hand, once formed, it should not be affected by additional adversity and thus provides the basis for maintaining psychological health [20]. However, this appears to contradict the repeated findings of worse mental and physical health in individuals that experienced early-life adversities and trauma [21, 22]. To better understand the role of SOC-R with respect to the relationship between early-life adversity and psychological health in older adults, we conducted cross-sectional [19] and longitudinal analyses with Sample 3 [23] in order to examine the potential moderating role of SOC-R. Cross-sectional analyses revealed a meaningful moderation effect of SOC-R on the association between childhood emotional neglect and current mental health. The study also provided initial evidence for a buffering effect of SOC-R against the detrimental impact of early-life adversity on mental health in later life in individuals with stronger SOC-R [19]. Longitudinal analyses (two assessments one year apart), partly replicated these findings, by showing a moderation effect for the SOC-R sub-scale ‘manageability’ (but not for the total SOC-R) on the relationship between chronic (recent) stress and mental health [23].

The initial validations of the newly constructed SOC-R are promising and suggest that the revised SOC scale is a valid and reliable measure to assess sense of coherence in the conducted studies with individuals at varying adversity levels and age stages. They further propose a moderating impact of the SOC-R on the association between early adversity and current mental health. Nonetheless, due to the relatively small sample size and non-representative nature of the previous sample compositions, generalization of these results is limited. We therefore aim to examine the psychometric properties of the revised SOC scale in a representative German sample. We hypothesized that the SOC-R would not be influenced by the socio-demographic characteristics age and gender. As Bachem and Maercker [14] found a positive association between education and the subscales ‘manageability’ and ‘reflection’, we hypothesized that SOC-R is influenced by level of education. Based on initial exploratory analyses by Mc Gee et al. [19] we set out to further examine the potential moderating role of SOC-R on the relationship between childhood adversity and mental health in a larger sample. We hypothesized that SOC-R would act as a moderator not only between childhood adversity (in particular emotional neglect) and mental health (depression and anxiety), but also between potentially traumatic events experienced during the lifetime and current mental health.

## Materials and methods

### Study design

The present study was part of a nationwide household survey conducted in Germany. The larger research endeavour of this survey study was the collective assessment of various aspects of the physical and mental well-being of a representative sample of the German resident population. In the complete survey, 23 different assessment units were included in the survey package (in addition to a demographic survey), comprising of single items (e.g. BMI, diabetes), item clusters (e.g. on migration background), and standardized instruments (e.g. assessing eating disorder, head ache, pain regions, or self-injurious thoughts). The current study focused on data collected on childhood maltreatment and trauma and lifetime traumatic events, depression and anxiety, and use of psychological resources (as described below). A representative sample of the general population was collected with the assistance of a demographic-consulting company (USUMA, Berlin, Germany), on behalf of the University of Leipzig. The study was conducted in accordance with the Declaration of Helsinki. It further fulfilled the ethical guidelines of the International Chamber of Commerce and of the European Society of Opinion and Marketing research. The study was approved by the ethics committee of the Medical Faculty of the University of Leipzig (297/16-ek).

### Participants

Eligible participants were individuals aged 14 years or older and had the ability to read and understand the German language. No additional inclusion or exclusion criteria were applied.

### Procedure

As part of the sampling procedure, a network was used consisting of 258 sample areas representative of the different regions in Germany. Within each regional area, households were then selected using random route procedure. The Kish-Selection-Grid technique was then used to sample individuals on the doorstep by randomly selecting one member from each household who fulfilled the inclusion criteria. From the 258 sample points, a total of 4902 households were selected for the study, of which 4838 were valid. A maximum of four attempts were made to contact the selected member of the household. Data collection took place from September to November 2016 and a total of 2510 face-to-face interviews were conducted (a participation rate of 51.9% of the valid addresses). Each participant was assessed once, with a study assistant present at each interview. Participation in the study was voluntary and responses were assessed anonymously. Participants were first informed about the study procedure and about the possibility of a later withdrawal of their consent. In the case of the participation of minors, a parent was additionally informed about the content of the study and the selection procedure. All participants provided informed consent. The consent received from the participants and/or guardians was verbal.

### Measures

In addition to the standardised questionnaires, participants completed a demographic survey, which collected data on age, gender, family status, living situation, education, employment status, and financial status.

#### Sense of coherence scale—Revised

The SOC-R scale [14] measures the way individuals perceive and integrate life experiences in order to maintain and develop health. It yields a total score and consists of 13 items rated on a five-point Likert scale. The SOC-R scale has three dimensions: *Manageability* (e.g., “One can always find a way to cope with painful things in life”; also, including one recoded item: “Difficult situations overstrain me”), *Reflection* (e.g., “Normally I can consider a situation from various perspectives”), and *Balance* (e.g., “In my thoughts and actions I take into account that things often have two sides: good and bad ones”). Response options range from 1 (not at all true) to 5 (extremely true). It is available in English and German, and initial results with the German version show adequate/good internal consistency of between α = .75 and .81 for the total scale, and poor to adequate internal consistency of between α = .57 and .77 for the three subscales. It has also been shown to have a high retest reliability coefficient of *r* = .85 over four weeks, and *r* = .74 over 15 months [14].

#### The Patient Health Questionnaire-4

The Patient Health Questionnaire-4 (PHQ-4, [24]) is used as a screening instrument for depression and anxiety. It consists of two items assessing core symptoms of depression (PHQ-2) and two items assessing core symptoms for generalized anxiety disorder (GAD-2). Participants are asked to consider the preceding two weeks when completing the instrument and response options range from 0 (not at all) to 3 (nearly every day). For each subscale, a score of 3 or greater is considered positive for screening purposes. The instrument shows adequate internal consistency of α = .78 for the total scale, α = .75 for the depression subscale, and good internal consistency α = .82 for the anxiety subscale [24].

#### Childhood Trauma Questionnaire

The Childhood Trauma Questionnaire (CTQ, [25]) assesses five types of trauma and maltreatment experienced during childhood: physical, sexual, and emotional abuse, and physical and emotional neglect. It consists of 28 items rated on a five-point Likert scale with response options ranging from 1 (never true) to 5 (very often true). The German version shows good internal consistency of α = .87 for the emotional abuse subscale, α = .80 for the physical abuse subscale, α = .89 for the sexual abuse subscale, α = .83 for the emotional neglect subscale, and, to a lesser extent, α = .55 for the physical neglect subscale [26].

#### Life Events Checklist

The Life Events Checklist for DSM-5 (LEC-5, [27, 28]) is used to screen for potentially traumatic events experienced in a participant’s lifetime. Comprised of 17 items, it assesses lifetime exposure to 16 events potentially associated with posttraumatic stress disorder (PTSD) or distress, and includes one item to assess any additional stressful event not captured in the previous 16 items. The LEC-5 generates the total number of events experienced personally, witnessed, learned about, and exposed to as part of your job. In the current study, total number of events experienced was used. Psychometric properties are currently not available for the LEC-5. However, the revisions from the original version of the LEC (LEC for DSM-IV) were minimal and the psychometric prosperities of the LEC-5 can be indirectly established based on its similarity to the original LEC [27]. The LEC for DSM-IV showed adequate test-retest reliability at both the item and total scale level over a one-week period. It also showed sufficient convergent validity with all but one item producing a kappa coefficient of .40 or higher [28].

### Statistical analysis

IBM Statistical Package for Social Sciences (SPSS) version 24.0 was used to analyse the data (IBM Corp., Armonk, N.Y., USA, [29]). There were less than 1% missing values for each instrument and Little’s missing completely at random (MCAR) test indicated that data in all but one instrument were MCAR [30]. Due to low percentage of missing data, values were replaced using the Expectation-Maximization algorithm [31]. The means of the participant on a subscale level were calculated for data not missing at random. Confirmatory Factor Analysis (CFA) assessed model fit using maximum likelihood estimation procedures. Good model fit was determined following recommendations by Hu and Bentler [32], Marsh et al. [33, 34], and Kline [35]: root mean square error of approximation (RMSEA) ≤ .06, standardised root mean square residual (SRMR) ≤ .08, comparative fit index (CFI) ≥ .95, and Tucker-Lewis index (TLI) ≥ .95. A non-significant chi-square (χ^2^) also indicated good model fit, but was used as a secondary fit index due to its sensitivity to large samples. The following cut-off criteria were used for factor loadings: > .71 = excellent, .63–.70 = very good, .55–.62 = good, .45–.54 = fair, .32–.44 = poor, and < .32 = not interpreted [36]. Internal consistency was assessed using Cronbach’s alpha (α), with alpha coefficients of ≥ .70 considered adequate and ≥ .80 considered good [37]. Pearson’s correlations were examined between the SOC-R scale and other psychological measures to test for discriminant validity. Following the cut-off criteria outlined by Cohen [38], correlations of .10 were considered small, .30 moderate, and .50 large. An independent t-test was used to test for differences in SOC-R between the two gender groups. A one-way between samples ANOVA was used to test for differences in SOC-R between the age groups and education levels. Additional post-hoc analyses (Gabriel’s test and the Games-Howell test) were conducted to further examine differences between the groups.

## Results

### Sample characteristics

The total sample consisted of 2510 participants, from which 109 multivariate outliers were removed. Due to data missing at the total scale or subscale level, an additional *n* = 28 participants were then excluded from the analyses. The final sample consisted of *N* = 2373 participants, with an age range of 14–94 years (*M* = 48.24 years; *SD* = 18.26). The sample was comprised of 1240 females (52.3%) and 1133 males. The majority of participants had completed secondary school education (53.3%) or primary school education (29.7%) and 10.2% had completed university-level education. Table 1 summarizes sample characteristics.

**Table 1:** Sample characteristics.

Sample Characteristics	Total (*N* = 2373)		Male		Female	
	**M**	***SD***	***n***	***%***	***n***	***%***
Age (years; age range = 14–94 years)	48.24	18.26				
Age groups:						
Youths (up to 18.9 years; *n* = 106)	16.25	1.26	56	47.2	50	52.8
Young adults (19.0–34.9 years; *n* = 536)	26.97	4.62	272	50.7	264	49.3
Middle age (35.0–49.9 years; *n* = 569)	42.17	4.44	251	44.1	318	55.9
Late-middle age (50.0–64.9 years; *n* = 639)	56.54	4.32	316	49.5	323	50.5
Older adults (65.0 years and older; *n* = 523)	73.01	6.07	238	45.5	285	54.5
	***n***	**%**				
Gender (% female)	1240	52.3	–	–	–	–
Education level: (*n* = 2367)						
Lower than primary school	51	2.1	24	47.1	27	52.9
Primary school	705	29.7	342	48.5	363	51.5
Secondary / High school	1265	53.3	583	46.1	682	53.9
Vocational training	103	4.3	53	51.5	50	48.5
University	243	10.2	129	53.1	114	46.9
Employment status: (*n* = 2354)						
Full-time	1030	43.4	655	57.8	375	30.2
Part-time	318	13.4	36	3.2	282	22.7
Voluntary work	24	1.0	3	.3	21	1.7
Currently unemployed	115	4.8	62	5.5	53	4.3
Retired	596	25.1	257	22.7	339	27.3
Not working	72	3.0	1	.1	71	5.7
Training	41	1.7	21	1.9	20	1.6
In education	158	6.7	89	7.9	69	5.6
Marital status: (*n* = 2364)						
Married	1099	46.3	531	46.9	568	45.8
Single	746	31.4	415	36.6	331	26.7
Divorced	320	13.5	143	12.6	177	14.3
Widowed	199	8.4	40	3.5	159	12.8

*Note*. *M* = mean; *n* = number; *SD* = standard deviation.

### Factorial structure of SOC-R

**Fig 1:**
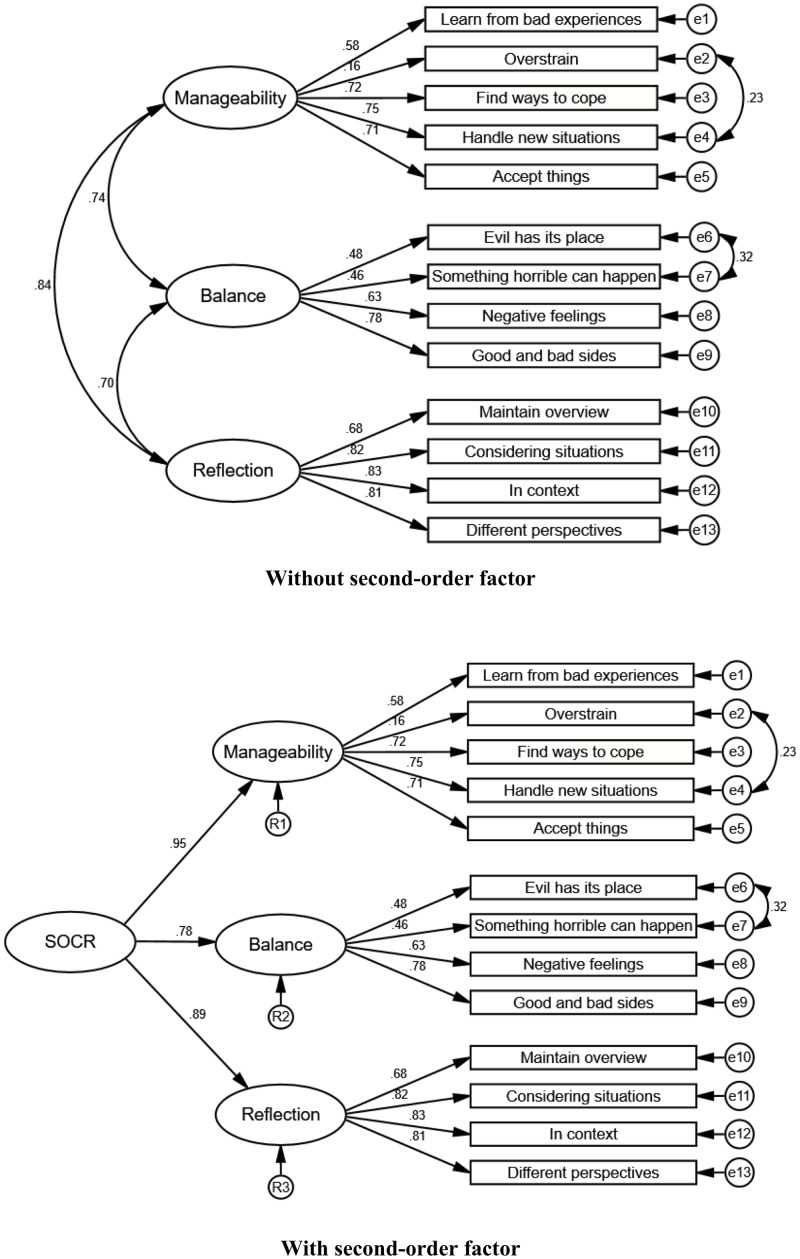
Factor structure of the modified three-factor model (with and without the second-order factor) with factor loadings and co-variances.

To examine the factor structure, one-factor and three-factor solutions were modelled using CFA. The fit statistics for the three CFA models are presented in Table 2. Fit indices indicated a poor model fit for the one-factor model (χ^2^ (65) = 1931.85, *p* < .01) with a RMSEA and 90% confidence interval of .110 [.106, .114] and an SRMR of .073. Poor model fit was also indicated by the CFI (.85) and TLI (.82). Fit indices showed improved model fit for the three-factor model (χ^2^ (62) = 987.12, *p* < .01), with the RMSEA of .079 [.075, .084] and SRMR (.056) both meeting the criteria for good model fit. However, better model fit was shown in the modified three-factor model (χ^2^ (60) = 683.49, p < .01), as indicated by the RMSEA (.066 [.062, .071]) and SRMR (.047). The CFI (.95) also indicated good model fit, with the TLI (.93) showing acceptable fit just below the cut-off criteria of ≥ .95. With regard to item loadings, most standardized factor loadings met the cut-off criteria of .32 (indicating an explained variance of 10%). Factor loadings ranged from fair to excellent with a range of .46 to .83, with the exception of item two on the manageability subscale (“Difficult situations overstrain me”), which showed a factor loading of .16. Errors for two items in the ‘balance’ subscale were co-varied following the model in Bachem and Maercker [14]. In addition, a high covariance of errors was also identified for two items in the ‘manageability’ subscale. Further inspection of these items (‘*6*. *Difficult situations overstrain me*’ and ‘*8*. *Due to my experiences in life I can handle new situations well*’) showed that the items were similarly worded (with one reverse-scored item), focusing on the manageability of different types of situations. According to research by Marsh [39] and Brown [40], such covariation may be a result of response style associated with the similar wording of the two items. These errors were therefore allowed to co-vary, which improved model fit. Moderately high correlations were shown between the three factors, indicating the presence of a higher-order factor. Therefore, “sense of coherence (SOC-R)” was modelled as a second-order factor with “manageability”, “balance”, and “reflection” as three first-order factors. Fit statistics remained the same as in the modified three-factor model and all factor loadings for the second-order factor loaded significantly on the three first-order factors within the excellent range (> .71). The factor structure of the modified three-factor model with and without the second-order factor is shown in Fig 1.

**Table 2:** Fit indices for the confirmatory factor analyses (CFA) models of the revised sense of coherence (SOC-R) scale.

Model	χ^2^ (*p* value)	*df*	RMSEA & CI	SRMR	CFI	TLI	AIC	BIC
1-factor model	1931.849 (*p* = .000)	65	.110 [.106, .114]	.073	.848	.817	1983.85	2133.92
3-factor model	987.115 (*p* = .000)	62	.079 [.075, .084]	.056	.925	.905	1045.12	1212.50
**3-factor model (modified)**	**683.487** **(*p* = .000)**	**60**	**.066** **[.062, .071]**	**.047**	**.949**	**.934**	**745.49**	**924.42**
**Second-order**								

*Note*. Best fitting model in bold.

AIC = Akaike information criterion; BIC = Bayesian information criterion; CFI = comparative fit index; CI = confidence interval; *df* = degree of freedom; *p* = probability; RMSEA = root mean square error of approximation; SRMR = standardised root mean square residual; TLI = Tucker-Lewis index; χ^2^ = significant chi-square.

### Internal consistency of the SOC-R

Reliability analyses showed a Cronbach’s alpha coefficient of α = .87 for the SOC-R total scale, indicating good internal consistency. The ‘reflection’ subscale also showed good internal consistency (.86), with the ‘manageability’ (α = .72) and ‘balance’ (α = .71) subscales showing adequate internal consistency.

### Discriminant validity of the SOC-R

Discriminant validity was demonstrated by significant negative correlations with small effect sizes between the SOC-R total scale and psychopathological constructs, such as anxiety (*r* = -.08, *p* < .01), depression (*r* = -.10, *p* < .01), and various indicators of childhood trauma and maltreatment (*p* < .01). Emotional neglect (*r* = -.27, *p* < .01) and physical neglect (*r* = -.25, *p* < .01) showed the largest correlations with SOC-R, although within the range of a small effect size. All SOC-R subscales showed significant or non-significant negative correlations with the psychopathological constructs, with the exception of the balance subscale, which showed significant, but low positive correlations with anxiety (*r* = .06, *p* < .01), depression (*r* = .05, *p* < .05), and emotional abuse (*r* = .04, *p* < .05). In addition, significant positive correlations with small effect sizes were shown between the LEC-5 and the total SOC-R scale (*r* = .10, *p* < .01), as well as with all SOC-R subscales (*p* < .01). See Table 3 for the correlation matrix.

**Table 3:** Discriminant correlations of revised sense of coherence (SOC-R) scale with psychopathological measures.

Measures	*M*	*SD*	SOC-R Total	Manageability	Balance	Reflection
SOC-R Total:	44.82	8.03	–			
Manageability	17.39	3.33	.86**	–		
Balance	12.83	3.16	.78**	.47**	–	
Reflection	14.60	3.10	.87**	.68**	.50**	–
Total burden on health:	1.23	1.94	–.10**	–.21**	.06**	–.08**
Anxiety	.61	1.02	–.08**	–.18**	.06**	–.08**
Depression	.62	1.05	–.10**	–.20**	.05*	–.08**
Childhood trauma and maltreatment:						
Emotional abuse	6.52	2.36	–.06**	–.12**	.04*	–.08**
Physical abuse	5.63	1.67	–.10**	–.11**	–.01	–.13**
Sexual abuse	5.36	1.21	–.08**	–.09**	–.004	–.10**
Emotional neglect	9.18	4.12	–.27**	–.27**	–.13**	–.29**
Physical neglect	7.53	2.77	–.25**	–.20**	–.16**	–.27**
Total adverse events experienced	.81	1.70	.13**	.10**	.10**	.11**

* p < .05, two-tailed;

** p < .01, two-tailed; significant results in bold.

**Instruments:** total burden on health = PHQ-4; childhood trauma = CTQ; total adverse events experienced = LEC-5.

*M* = mean; *SD* = standard deviation; SOC-R = revised sense of coherence (SOC-R) scale.

### The role of gender within the SOC-R

There were balanced proportions of males (*n* = 1113) and females (*n* = 1240) in the sample who completed the SOC-R scale. An independent t-test showed that the difference (.657, 95% CI [.010, 1.303]) in the SOC-R total score between gender was marginally significant, (*t* (2371) = 1.99, *p* = .05), with males (*M* = 45.16) showing slightly higher scores than females (*M* = 44.50). However, this was with a very small effect size (*d* = .082) suggesting a negligible difference. The ‘manageability’ subscale also showed a significant difference (.440, 95% CI [.173, .708]) in SOC-R between the two genders, (*t* (2371) = 3.23, *p* < .01), with males (*M* = 17.62) showing marginally higher scores than females (*M* = 17.18). However, this was also with a small effect size (*d* = .133). Non-significant results with small effect sizes were shown for both the ‘balance’ and ‘reflection’ subscales.

### The role of age within the SOC-R

**Fig 2:**
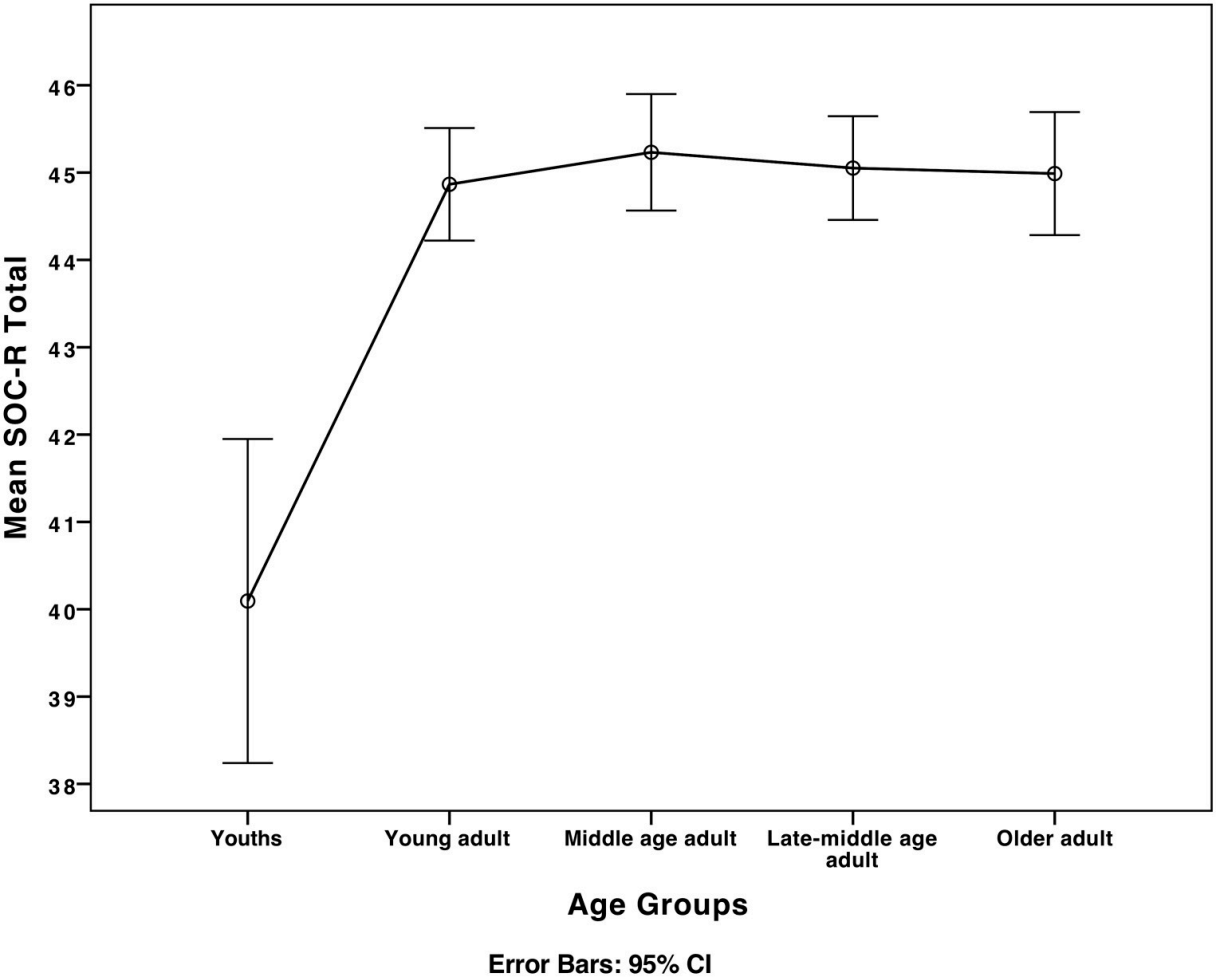
Plot of mean differences between age groups in revised sense of coherence (SOC-R) total.

Participants were divided into age groups to examine differences in SOC-R between the groups (see Table 1 for sample characteristics of the different age groups). ANOVA results showed that SOC-R differed significantly between the age groups, (*F* (4, 2368) = 9.91, *p* < .01, η^2^ = .02). See Fig 2 for the plot of differences in SOC-R between age groups (see Fig 2). Similar results were shown for the ‘manageability’ subscale (*F* (4, 2368) = 11.89, *p* < .01, η^2^ = .02), ‘balance’ subscale (*F* (4, 2368) = 5.32, *p* < .01, η^2^ = .01), and ‘reflection’ subscale (*F* (4, 2368) = 7.32, *p* < .01, η^2^ = .01). Additional post-hoc analyses showed that significant differences were found between the youths age group and all other age groups for both the SOC-R total scale (*p* < .01) and all the subscales ‘manageability’ (*p* < .01), ‘balance’ (*p* < .05), and ‘reflection’ (*p* < .01).

### The role of education within the SOC-R

Differences in SOC-R were examined between groups of participants with differing education levels (see Table 1 for the sample characteristics of the different education groups). Results showed that SOC-R differed significantly between the education levels, (*F* (4, 2362) = 13.98, *p* < .01, η^2^ = .02). Similar results were shown for the ‘manageability’ subscale (*F* (4, 2362) = 10.14, *p* < .01, η^2^ = .02), ‘balance’ subscale (*F* (4, 2362) = 4.90, *p* < .01, η^2^ = .01), and ‘reflection’ subscale (*F* (4, 2362) = 17.65, *p* < .01, η^2^ = .03). Additional post-hoc analyses showed that significant differences in SOC-R were found between the university education group and the lower than primary school education group (*p* < .01), the primary school education group (*p* < .01), and the secondary/high school education group (*p* < .01). A marginally significant difference was also shown between the vocational training group and the primary school education group (*p* = .05). Similar results were shown for the subscales, with additional significant differences found for the ‘manageability’ subscale between the vocational training group and the secondary/high school education group (*p* < .05); and for the ‘reflection’ subscale between the secondary/high school education group and the primary school education group (*p* < .05). Overall, SOC-R total and subscale scores were higher for more educated participants than for less educated participants.

### The moderating role of SOC-R on life adversities and depression

Moderation analysis was used to examine the influence of SOC-R on the relationship between adversities (in the form of childhood trauma and maltreatment and lifetime traumatic events) and anxiety and depression. In relation to childhood trauma and maltreatment, participants reported highest levels of emotional neglect (*M* = 9.18) followed by physical neglect (*M* = 7.53), emotional abuse (*M* = 6.52), physical abuse (*M* = 5.63), and sexual abuse (*M* = 5.36), respectively. With regard to anxiety, no significant interaction effects were shown for the CTQ subscales and total SOC-R.

**Fig 3:**
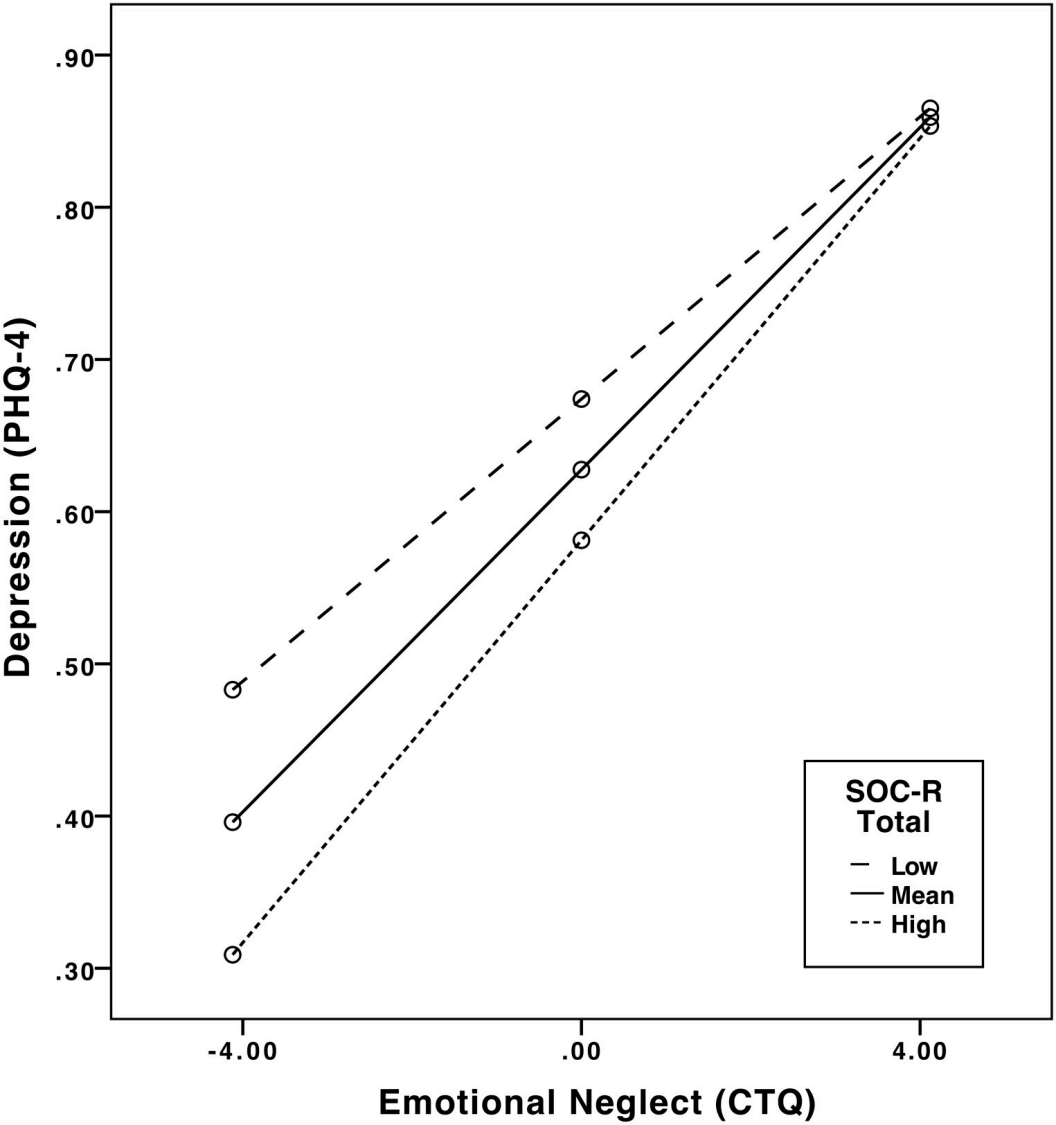
Plot of the regression of emotional neglect on depression at different levels of the moderator (SOC-R). Note: High and low levels refer to one standard deviation above and below the mean value of the moderator (i.e. SOC-R).

With regard to depression, a significant interaction effect was shown for emotional neglect and total SOC-R (*b* = .001, 95% CI [.000, .002], *t* = 2.29, *p* < .05), indicating that SOC-R significantly moderates the relationship between emotional neglect and depression. A significant positive relationship was observed between emotional neglect and depression at low levels of SOC-R (*b* = .046, 95% CI [.034, .059], *t* = 7.25, *p* < .01), mean levels of SOC-R (*b* = .056, 95% CI [.044, .068], *t* = 9.34, *p* < .01), and high levels of SOC-R (*b* = .066, 95% CI [.050, .082], *t* = 7.97, *p* < .01), suggesting that emotional neglect was significantly associated with depression at all levels of SOC-R. Low levels refer to one standard deviation below the mean value of the moderator (i.e. SOC-R) and high levels refer to one standard deviation above the mean value of the moderator. However, visual inspection of the interaction plot showed that individuals with high SOC-R had lower scores of depression in comparison to individuals with mean and low SOC-R, particularly at mean and low levels of emotional neglect. See Fig 3 for the plot of the significant interaction and moderation effects. No significant interaction effects were shown with the other CTQ subscales.

**Fig 4:**
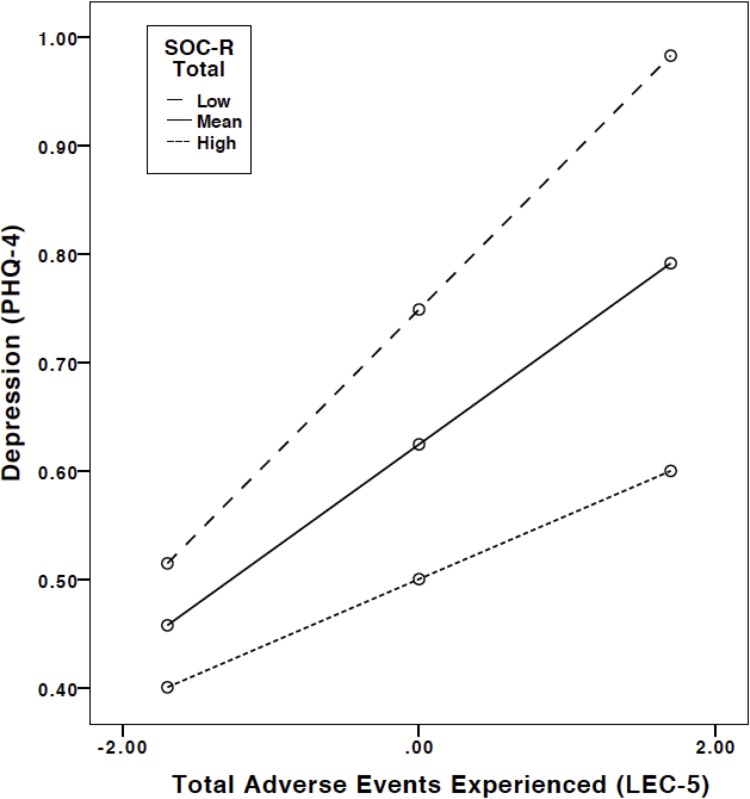
Plot of the regression of total adverse events experienced on depression at different levels of the moderator (SOC-R). Note: High and low levels refer to one standard deviation above and below the mean value of the moderator (i.e. SOC-R).

In relation to cumulative lifetime traumatic events, no significant interaction effect was shown for anxiety and total SOC-R. However, a significant interaction effect was shown for depression and total SOC-R (*b* = –.005, 95% CI [–.009,–.001], *t* = –2.37, *p* < .05), indicating that SOC-R significantly moderates the relationship between lifetime traumatic events and depression. A significant positive relationship was observed between lifetime traumatic events experienced and depression at low levels of SOC-R (*b* = .138, 95% CI [.078, .198], *t* = 4.52, *p* < .01), mean levels of SOC-R (*b* = .098, 95% CI [.059, .138], *t* = 4.92, *p* < .01), and high levels of SOC-R (*b* = .059, 95% CI [.018, .099], *t* = 2.85, *p* < .01). However, the decreasing effect indicates a buffering effect in which increases in total SOC-R decrease the effect of adverse events on depression. Visual inspection of the interaction plot supports this, as individuals with high SOC-R had lower scores of depression in comparison to individuals with mean and low SOC-R, particularly at high levels of traumatic events. See Fig 4 for the plot of the significant interaction and moderation effects.

## Discussion

The revised SOC scale has previously been validated in non-representative samples biased with respect to level of adversity, gender, and age. It was therefore the aim of the current study to evaluate the revised SOC scale to examine its psychometric properties in a population-based representative sample. CFA confirmed the previously identified three-factor structure of the SOC-R with the dimensions ‘manageability’, ‘balance’, and ‘reflection’, with acceptable model fit indices. Reliability analyses revealed good internal consistency. Discriminant validity indicates that the SOC-R is an independent construct with regard to examined measures of psychopathology and childhood trauma and maltreatment. Gender groups differed marginally in the total SOC-R scale and significantly in the subscale ‘manageability’. With regard to age, we found a meaningful difference between the youngest age group and all other age groups in the SOC-R total scale, as well as all subscales. Higher education was found to be related to higher levels of SOC-R. Moderation analyses revealed that SOC-R may exert a protective influence on depression at all levels of cumulative lifetime traumatic events (low, mean, high), as well as on low and mean levels of childhood maltreatment (i.e. emotional neglect).

The previously identified three-factor structure of the SOC-R [14, 19] with the dimensions ‘manageability’, ‘balance’, and ‘reflection’ were replicated in the current study. Best model fit was found in the modified model, in which certain error variances were allowed to co-vary. Item loadings were mostly found to be significant, with the exception of item 2 of the ‘manageability’ subscale (i.e. item 6 of the whole SOC-R scale: “Difficult situations overstrain me”). This item is the only reverse-coded item in the whole scale, which might be a reason for its low factor loading. The implied opposite of being ‘overstrained’ by a difficult situation may not truly capture the core meaning of the ‘manageability’ subscale (i.e. the ability to come to terms with difficult situations). It may be advisable to rephrase this particular item into “I perceive difficult situations as challenges to be / I can overcome”, for instance. Item 2 of the ‘balance’ subscale (i.e. item 3 of the whole scale: “I know that I could suddenly experience something really horrible or shocking”), showed a poor factor loading in Sample 3 [19]. However, this item significantly loaded on the ‘balance’ subscale and also met cut-off criteria, indicating no issues with that particular item in the current study. Due to the moderately high correlations between the three subscales and based on the research by Bachem and Maercker [14], the SOC-R construct was included in the CFA model as a higher-order factor [35, 41]. This resulted in significant factor loadings, suggesting that the SOC-R construct consists of three sub-constructs (i.e. the three subscale factors). This structure supports the model proposed by Bachem and Maercker [14]. In sum, with this study in a representative sample we were able to confirm the three-factor structure which composes SOC-R. It may be necessary to reword item 6 of the SOC-R scale in order to address the low factor loadings of this item.

Reliability analyses revealed good internal consistency for the total scale. Good internal consistency was also shown for the ‘reflection’ subscale and adequate internal consistency for the ‘manageability’ and ‘balance’ subscales. We can conclude from this that a repeatedly found weakness of the instrument, i.e. the less than optimal internal consistency for the ‘balance’ subscale [14, 19], might have been caused by sample size and / or particular sample characteristics and is not due to an inherent lack of internal consistency of the revised scale. Given the fact that data was assessed at only one time point, we cannot make any conclusions about the stability of the instrument. The longest observation period so far is the time-frame of 15 months in the study by Bachem and Maercker [14]. Future studies are needed to test the stability of the measure over time.

Discriminant validity analyses revealed meaningful significant negative correlations of the SOC-R total score, as well as the subscales ‘manageability’ and ‘reflection’, with measures of psychopathology, i.e. total health burden and depression and anxiety, with small effect sizes. The ‘balance’ subscale was positively associated with those measures, with small effect sizes. These results indicate that the SOC-R shows some relation to psychopathology, but the small effect sizes support the idea that the SOC-R does not simply constitute an inverse measure of psychopathology [10], particularly in view of the positive correlation with the ‘balance’ subscale and the positive correlation between the LEC-5 and total SOC-R, as well as with the subscales.

Gender was shown to have a marginal impact on the SOC-R total scale. It may be assumed that the significant difference between males and females on the subscale ‘manageability’ might have contributed to the overall marginal significant difference between genders on the SOC-R scale. Taking into consideration the small and negligible difference evidenced by the very small effect size, we may cautiously conclude that SOC-R total score may not be affected by gender. The significant difference in the ‘manageability’ subscale, with males showing higher levels, was also very small in size. It may therefore be that this particular subscale is affected by the influence of gender. It can only be speculated why the ‘manageability’ subscale is more affected by gender in comparison to the other subscales. One possible explanation for this may be the fact that the ‘manageability’ subscale mostly involves coping related items/aspects; and previous research suggests that males and females appear to differ with regard to applied coping strategies [42, 43]. Future studies are needed to specifically examine the influence of gender on the subscales of the SOC-R.

We found a significant difference between the age group ‘youths’, including participants up to 18 years, and all other age groups for both the SOC-R total scale and all subscales. This finding implies an impact of age (phase) on the SOC-R scale. In the ‘youth’ age group, SOC may not be as well-developed as in the older age groups, which might have caused the lower levels. The non-significant differences in the SOC-R between all older age groups suggest stability in the measure after initial formation. While this finding partially parallels Antonovsky’s theoretical assumption [3] of SOC development over the life course (i.e. increase in SOC until young adulthood, stability until retirement, and subsequent decline) [5], it is not in accordance with cross-sectional [44] and longitudinal studies [45] that suggest an increase in SOC in the process of aging (see also, [46]). Given the cross-sectional (i.e. interindividual) assessment of the SOC-R over different age-groups in the current study, we may not make final conclusions about the trajectories of the revised sense of coherence conceptualization and scale across the lifespan. Future studies should conduct follow-up analyses with participants over longer periods of time to deduce intraindividual stability of the SOC-R.

Higher education was found to be related to higher scores in the SOC-R total scale as well as in all three subscales. This is in line with and expands previous findings reported by Bachem and Maercker [14], who found a positive association between the subscales ‘reflection’ and ‘manageability’ and level of education. However, Bachem and Maercker [14] found this positive relationship only in the healthy control sample and not in the bereaved sample which was interpreted as a weaker impact of education in the context of adversity.

Moderation analyses revealed that in the case of lifetime traumatic events, higher SOC-R was found to lower the association between these events and depression. Independent of the number of traumatic events (i.e. low, mean, high), individuals with high SOC-R showed lower levels of depression than individuals with lower SOC-R scores. This indicates a potentially protective effect of the SOC-R on the association between traumatic events and depression. A different picture emerged when looking at childhood trauma and maltreatment: Moderation analyses with the CTQ indicated that while high levels of SOC-R seemed to exert a protective effect on depression at low and mean levels of emotional neglect, this effect disappeared at high levels of emotional neglect. Due to the sensitive period of childhood with regard to psychoneuroendocrinological development, adversities may exert a more pronounced impact on the mental health of the affected individual, in comparison to adversities experienced later in life (see for instance, [21, 47, 48]). In the face of high levels of adversity, protective influences may therefore be generally less effective. However, this is a speculative conclusion.

A major strength of the current study is the representative nature of the sample. An additional strength is the inclusion of various measures of trauma and maltreatment, covering not only the sensitive period of childhood but also lifetime traumatic events.

### Limitations of the study

One point that must be acknowledged, given the revised nature of the SOC-R scale, is the comparability of SOC-R to Antonovsky’s [1] original SOC scale. While Antonovsky’s original SOC deals with the predictability and comprehensibility of life [1, 3], the SOC-R concept takes a more neutral position of dealing with the ambiguity of life experiences [14]. Nevertheless, both concepts similarly refer to a method for dealing with life experiences in a way that facilitates the successful overcoming of stressors. SOC-R may therefore be viewed as a refined SOC construct. In fact, Antonovsky predicted such a revision: “… there is no doubt in my mind that in 5 years or so, sufficient evidence will have accumulated to provide the basis for a second generation SOC scale” (p. 732, [4]). The SOC scale was revised by Bachem and Maercker [14] in order to build on the original SOC and improve the psychometric properties. In particular, SOC-R has been shown to be more clearly differentiated than the original SOC, as it showed less overlap with psychopathological constructs, such as depression and anxiety (in the current study), as well as with self-efficacy and the personality traits of neuroticism and optimism [14, 19]. Findings for this revised scale support SOC-R to be a more refined and better delineated sense of coherence. However, research with the SOC-R is still at the early stages and further studies are required to replicate this.

Additionally, while the discriminant validity hints at the refinement of the scale, future studies would benefit from further assessing the convergent validity of the SOC-R. For instance, previous studies suggest small positive associations between SOC-R and the personality trait of optimism [14, 19]. However, as SOC-R is proposed to facilitate the appropriate use of recourses, it may therefore be viewed as a mediatory construct, which may be more useful for targeting in psychotherapeutic and clinical settings, in comparison to more stable dispositions (e.g., personality). This is supported by the moderating role of SOC-R shown in the current study. Nevertheless, further research should assess convergent validity and incremental validity with constructs representing basic dispositions, such as the Big Five or HEXACO personality traits or intelligence.

Our results must be interpreted in the view of the further limiting factors: while the PHQ-4 is a broadly used instrument for depression and anxiety, it constitutes a screening, rather than a diagnostic measure. Our results should be replicated in studies applying established diagnostic instruments for the assessment of depression and anxiety. In addition, the cross-sectional design of the current study can be regarded as limiting factor as it does not allow the intraindividual development to be assessed with regard to the SOC-R over time. Finally, as data was assessed using self-report measures, future studies should include objective measures to exclude the possibility that findings were methodologically biased.

In conclusion, with the current study we were able to further support the psychometric properties of the revised SOC scale. Furthermore, we showed the moderating effect of SOC-R on the relationship between adversity (particularly cumulative lifetime traumatic events) and depression. This further emphasizes the necessity of considering salutogenic influences to better understand interindividual responses to childhood trauma and maltreatment.

## Supporting information

S1 Supporting information Raw data file. (SAV)

## References

[1] Antonovsky A. Health, stress, and coping. 1979.

[2] Mittelmark MB, Bauer GF. The Meanings of Salutogenesis In: Mittelmark MB, Sagy S, Eriksson M, Bauer GF, Pelikan JM, Lindstrom B, et al, editors. The Handbook of Salutogenesis. Cham (CH): Springer Copyright 2017, The Author(s). 2017 p. 7–13.

[3] Antonovsky A. Unraveling the mystery of health: How people manage stress and stay well: Jossey-Bass; 1987.

[4] Antonovsky A. The structure and properties of the sense of coherence scale. Social science & medicine (1982). 1993;36(6):725–33. Epub 1993/03/01. . 8480217 10.1016/0277-9536(93)90033-z

[5] Eriksson M, Mittelmark MB. The Sense of Coherence and Its Measurement In: Mittelmark MB, Sagy S, Eriksson M, Bauer GF, Pelikan JM, Lindstrom B, et al, editors. The Handbook of Salutogenesis. Cham (CH): Springer Copyright 2017, The Author(s). 2017 p. 97–106.

[6] Rajesh G, Eriksson M, Pai K, Seemanthini S, Naik DG, Rao A. The validity and reliability of the Sense of Coherence scale among Indian university students. Global health promotion. 2016;23(4):16–26. Epub 2015/04/22. 10.1177/1757975915572691 . 25897012

[7] Lerdal A, Opheim R, Gay CL, Moum B, Fagermoen MS, Kottorp A. Psychometric limitations of the 13-item Sense of Coherence Scale assessed by Rasch analysis. BMC psychology. 2017;5(1):18 10.1186/s40359-017-0187-y 28595651 PMC5465532

[8] Feldt T, Lintula H, Suominen S, Koskenvuo M, Vahtera J, Kivimaki M. Structural validity and temporal stability of the 13-item sense of coherence scale: prospective evidence from the population-based HeSSup study. Quality of life research: an international journal of quality of life aspects of treatment, care and rehabilitation. 2007;16(3):483–93. Epub 2006/11/09. 10.1007/s11136-006-9130-z . 17091360

[9] Zimprich D, Allemand M, Hornung R. Measurement invariance of the abridged sense of coherence scale in adolescents. European Journal of Psychological Assessment. 2006;22(4):280–7.

[10] Gruszczynska E. What is measured by the Orientation to Life Questionnaire? Construct validity of the instrument for the Sense of Coherence measurement. Polish Psychological Bulletin. 2006;37(2):74.

[11] Eriksson M, Lindstrom B. Antonovsky’s sense of coherence scale and the relation with health: a systematic review. Journal of epidemiology and community health. 2006;60(5):376–81. Epub 2006/04/15. 10.1136/jech.2005.041616 16614325 PMC2563977

[12] Volanen SM, Suominen S, Lahelma E, Koskenvuo M, Silventoinen K. Negative life events and stability of sense of coherence: a five-year follow-up study of Finnish women and men. Scandinavian journal of psychology. 2007;48(5):433–41. Epub 2007/09/20. 10.1111/j.1467-9450.2007.00598.x . 17877558

[13] Caap-Ahlgren M, Dehlin O. Sense of coherence is a sensitive measure for changes in subjects with Parkinson’s disease during 1 year. Scandinavian journal of caring sciences. 2004;18(2):154–9. Epub 2004/05/19. 10.1111/j.1471-6712.2004.00248.x . 15147478

[14] Bachem R, Maercker A. Development and psychometric evaluation of a revised sense of coherence scale. European Journal of Psychological Assessment. 2016.

[15] Rutter M. Resilience as a dynamic concept. Development and psychopathology. 2012;24(2):335–44. 10.1017/S0954579412000028 22559117

[16] Masten AS. Ordinary magic: Resilience processes in development. American psychologist. 2001;56(3):227 11315249 10.1037//0003-066x.56.3.227

[17] Rutter M. Implications of resilience concepts for scientific understanding. Annals of the New York Academy of Sciences. 2006;1094(1):1–12. 10.1196/annals.1376.002 17347337

[18] Bonanno GA. Loss, trauma, and human resilience: Have we underestimated the human capacity to thrive after extremely aversive events? American psychologist. 2004;59(1):20 10.1037/0003-066X.59.1.20 14736317

[19] Mc Gee SL, Holtge J, Maercker A, Thoma MV. Evaluation of the revised Sense of Coherence scale in a sample of older adults: A means to assess resilience aspects. Aging Ment Health. 2017:1–10. Epub 2017/08/12. 10.1080/13607863.2017.1364348 . 28799415

[20] Antonovsky A. The salutogenic model as a theory to guide health promotion. Health promotion international. 1996;11(1):11–8.

[21] Kessler RC, McLaughlin KA, Green JG, Gruber MJ, Sampson NA, Zaslavsky AM, et al Childhood adversities and adult psychopathology in the WHO World Mental Health Surveys. The British Journal of Psychiatry. 2010;197(5):378–85. 10.1192/bjp.bp.110.080499 21037215 PMC2966503

[22] Krammer S, Kleim B, Simmen-Janevska K, Maercker A. Childhood trauma and complex posttraumatic stress disorder symptoms in older adults: a study of direct effects and social-interpersonal factors as potential mediators. Journal of Trauma & Dissociation. 2016;17(5):593–607. 26011396 10.1080/15299732.2014.991861

[23] Mc Gee SL, Höltge J, Maercker A, Thoma MV. Sense of coherence and stress-related resilience: Investigating the mediating and moderating mechanisms in the development of resilience following stress or adversity. Frontiers in Psychiatry. 2018;9:378 10.3389/fpsyt.2018.00378 30186189 PMC6110848

[24] Löwe B, Wahl I, Rose M, Spitzer C, Glaesmer H, Wingenfeld K, et al A 4-item measure of depression and anxiety: validation and standardization of the Patient Health Questionnaire-4 (PHQ-4) in the general population. Journal of affective disorders. 2010;122(1):86–95. 19616305 10.1016/j.jad.2009.06.019

[25] Bernstein DP, Fink L. Childhood trauma questionnaire: A retrospective self-report: Manual: Psychological Corporation; 1998.

[26] Klinitzke G, Romppel M, Häuser W, Brähler E, Glaesmer H. Die deutsche Version des Childhood Trauma Questionnaire (CTQ)–psychometrische Eigenschaften in einer bevölkerungsrepräsentativen Stichprobe. PPmP-Psychotherapie· Psychosomatik· Medizinische Psychologie. 2012;62(02):47–51. 10.1055/s-0031-1295495 22203470

[27] Weathers F, Blake D, Schnurr P, Kaloupek D, Marx B, Keane T. The life events checklist for DSM-5 (LEC-5). Instrument available from the National Center for PTSD at www.ptsd.va.gov. 2013.

[28] Gray MJ, Litz BT, Hsu JL, Lombardo TW. Psychometric properties of the life events checklist. Assessment. 2004;11(4):330–41. 10.1177/1073191104269954 15486169

[29] Corp. I. IBM SPSS Statistics for Macintosh, Version 24.0. Released 2016. Armonk, NY, USA: IBM Corp.; 2016.

[30] Little RJ. A test of missing completely at random for multivariate data with missing values. Journal of the American Statistical Association. 1988;83(404):1198–202.

[31] Dempster AP, Laird NM, Rubin DB. Maximum likelihood from incomplete data via the EM algorithm. Journal of the royal statistical society Series B (methodological). 1977:1–38.

[32] Lt Hu, Bentler PM. Cutoff criteria for fit indexes in covariance structure analysis: Conventional criteria versus new alternatives. Structural equation modeling: a multidisciplinary journal. 1999;6(1):1–55.

[33] Marsh HW, Hau K-T, Wen Z. In search of golden rules: Comment on hypothesis-testing approaches to setting cutoff values for fit indexes and dangers in overgeneralizing Hu and Bentler’s (1999) findings. Structural equation modeling. 2004;11(3):320–41.

[34] Marsh HW, Lüdtke O, Muthén B, Asparouhov T, Morin AJ, Trautwein U, et al A new look at the big five factor structure through exploratory structural equation modeling. Psychological assessment. 2010;22(3):471 10.1037/a0019227 20822261

[35] Kline R. Principles and Practice of Structural Equation Modeling, 3rd edn Guilford Press New York 2011.

[36] Tabachnick B. BG Tabachnick, LS Fidell. Using multivariate statistics. 2013;6.

[37] Cronbach LJ. Coefficient alpha and the internal structure of tests. psychometrika. 1951;16(3):297–334.

[38] Cohen J. Statistical power analysis for the behavioral sciences. Hillsdale, NJ: L. Lawrence Earlbaum Associates 1988;2.

[39] Marsh HW. Positive and negative global self-esteem: A substantively meaningful distinction or artifactors? Journal of personality and social psychology. 1996;70(4):810 8636900 10.1037//0022-3514.70.4.810

[40] Brown T. Methodology in the social sciences Confirmatory factor analysis for applied research (2nd ed). New York, NY, US: Guilford Press; 2015.

[41] Rindskopf D, Rose T. Some theory and applications of confirmatory second-order factor analysis. Multivariate behavioral research. 1988;23(1):51–67. 10.1207/s15327906mbr2301_3 26782257

[42] Matud MP. Gender differences in stress and coping styles. Personality and individual differences. 2004;37(7):1401–15.

[43] Tamres LK, Janicki D, Helgeson VS. Sex differences in coping behavior: A meta-analytic review and an examination of relative coping. Personality and social psychology review. 2002;6(1):2–30.

[44] Nilsson KW, Leppert J, Simonsson B, Starrin B. Sense of coherence (SOC) and psychological well-being (GHQ): Improvement with age. Journal of Epidemiology & Community Health. 2009:jech. 2008.081174. 10.1136/jech.2008.081174 19692734

[45] Lövheim H, Graneheim UH, Jonsén E, Strandberg G, Lundman B. Changes in sense of coherence in old age–a 5‐year follow‐up of the Umeå 85+ study. Scandinavian journal of caring sciences. 2013;27(1):13–9. 10.1111/j.1471-6712.2012.00988.x 22462766

[46] Koelen M, Eriksson M, Cattan M. Older people, sense of coherence and community The Handbook of Salutogenesis: Springer; 2017 p. 137–49. 28590647

[47] Heim C, Nemeroff CB. The role of childhood trauma in the neurobiology of mood and anxiety disorders: preclinical and clinical studies. Biological psychiatry. 2001;49(12):1023–39. 11430844 10.1016/s0006-3223(01)01157-x

[48] Heim C, Newport DJ, Mletzko T, Miller AH, Nemeroff CB. The link between childhood trauma and depression: insights from HPA axis studies in humans. Psychoneuroendocrinology. 2008;33(6):693–710. 10.1016/j.psyneuen.2008.03.008 18602762

